# Gastroprotective Effect of Freeze Dried Stripped Snakehead Fish (*Channa striata* Bloch.) Aqueous Extract against Aspirin Induced Ulcerogenesis in Pylorus Ligated Rats

**DOI:** 10.1155/2014/327606

**Published:** 2014-05-29

**Authors:** Mohammed Safwan Ali Khan, Abdul Manan Mat Jais, Javeed Hussain, Faiza Siddiqua, A. Gopala Reddy, P. Shivakumar, D. Madhuri

**Affiliations:** ^1^Pharmacology & Toxicology Laboratory, Department of Biomedical Sciences, Faculty of Medicine and Health Sciences, University Putra Malaysia, 43400 Serdang, Selangor Darul Ehsan, Malaysia; ^2^Natural Products Research Laboratory and Department of Pharmacology, Anwarul Uloom College of Pharmacy, New Mallepally, Hyderabad, Andhra Pradesh 500 001, India; ^3^Department of Pharmacology & Toxicology, College of Veterinary Science, Acharya N.G. Ranga Agricultural University, Rajendra Nagar, Hyderabad, Andhra Pradesh 500 030, India; ^4^Department of Pathology, College of Veterinary Science, Acharya N.G. Ranga Agricultural University, Rajendra Nagar, Hyderabad, Andhra Pradesh 500 030, India

## Abstract

*Channa striata* (Bloch.) is a fresh water fish belonging to the family Channidae. The stripped snakehead fish possesses wide range of medicinal properties. In view of traditional use of *C. striata* for wound healing, the present study was undertaken to investigate the beneficial effects of orally administered freeze dried aqueous extract of *Channa striata* (AECS) in experimentally induced gastric ulcers in Wistar rats. Aspirin induced ulcerogenesis in pyloric ligation model was used for the assessment of antiulcer activity and Ranitidine (50 mg/kg) was employed as the standard drug. The various gastric parameters like volume of gastric juice, pH, free and total acidities, ulcer index, and levels of antioxidant enzymes like catalase, superoxide dismutase, and lipid peroxidation marker malondialdehyde were determined. AECS at concentrations of 40% and 50% w/v significantly decreased the volume of gastric juice and increased the levels of catalase while considerable decrease in free and total acidities and increase in superoxide dismutase were observed with the treatment of standard drug and AECS (50% w/v). All the test doses of AECS markedly decreased ulcer index and malondialdehyde compared to the standard drug whereas AECS 30% w/v did not alter volume of gastric juice, pH, free and total acidities, catalase, and superoxide dismutase. From these findings, it can be concluded that AECS is devoid of acid neutralizing effects at lower doses and possesses antisecretory and antiulcer activities and this could be related to its antioxidant mechanism.

## 1. Introduction

*Channa striata *(Bloch.) is commonly known as stripped snakehead fish and Haruan in Malay.* C. striata* is indigenous to Malaysia. It is a tropical and air breathing carnivorous freshwater fish from the family Channidae. There are all together 30 species in the family reported around the world and eight of them are found in Malaysia. Members of the family are also found in all ASEAN countries, namely, Myanmar, Thailand, Laos, Cambodia, Vietnam, Brunei, Philippines, Indonesia, and Singapore [1].* C. striata* is wild species found in small rivers, lakes, pools, and shallow water bodies and in its natural habitat. It can survive in harsh environments with low dissolved oxygen and high ammonia [2].* C. striata* contains all the essential amino and fatty acids required for wound healing [3, 4]; the fatty acid content of this fish is rich in arachidonic acid (ARA) [5]. Mohd Shafri and Mat Jais reviewed in detail the description, habitat, traditional therapeutic uses, chemical composition, and scientifically proven medicinal properties [6]. The fish is reported to possess antinociceptive [7, 8], antipyretic [9], antidepressant [10, 11], anti-inflammatory [12], antifungal [13], antimicrobial [14], antiosteoarthritic [15], neuroregenerative, and restorative [16] properties and enhances wound healing process [17–19]. In view of traditional and scientific reports on wound healing potential of* C. striata *and presence of wide range of bioactive components, the present study was undertaken to investigate antiulcer activity.

## 2. Material and Methods 

### 2.1. Preparation of Aqueous Extract of* Channa striata* (Bloch.) and Freeze Drying

Adult* Channa striata* (Bloch.) of medium size (total length of fish 20.60–44.10 cm), weighing 250–400 g/fish, were collected in November 2011 from Kuala Terengganu, Terengganu District (N 5°19′29.40′′ and E 103°08′27.29′′) from Peninsular Malaysia. The temperature was 28–33°C with pH between 5.60 and 8.20, salinity 0 ppt, dissolved oxygen in the range of 2.00–11.00 mg/L, turbidity in the range of 1.00–18.00 NTU, and conductivity 0.54-0.55 mS/cm. The fishes were verified by Terengganu Fisheries Department, Ministry of Agriculture, Malaysia. Fillets were obtained by carefully cutting the fish lengthwise along backbone to gain maximum amount of flesh. The whole fish fillet extract was prepared by using pressure cooker set at 100°C for 30 min. The fresh boneless fillet was weighed and placed on a stainless steel wire mesh mounted on a stainless steel tripod in the pressure cooker. Fish fillet and distilled water were added in volume ratio of 1 : 2. The extract was obtained through steaming. At the end of the extraction procedure, the fillet was discarded while collecting the liquid extract using Whatman no. 1 filter paper. The obtained extract was freeze dried using a freeze drier and stored at 20°C until use [7, 8].

### 2.2. Drugs and Chemicals

Chloroform AR, phenolphthalein pH indicator solution, and sodium hydroxide pellets were procured from SD Fine-Chem Limited, Mumbai, while pure aspirin was obtained from Divis Laboratories, Hyderabad. Ranitidine (as Rantac 150 mg) was from J.B Chemicals and Pharmaceuticals, Mumbai, while surgical spirit was obtained from Kakatiya Pharma, Hyderabad. Topfer's reagent and distilled water were obtained from Nice Chemicals, China, and Stangen Fine Chemicals, Hyderabad, respectively. All chemicals were used without further purification.

### 2.3. Animals

For the evaluation of the antiulcer property, male albino Wistar rats weighing 160–200 g were used. The study was conducted after the approval of protocol by Institutional Animal Ethical Committee (IAEC). The animals were maintained under standard conditions, that is, housed in polypropylene cages and maintained at a temperature 27 ± 2°C, relative humidity 65 ± 10% under 12-hour light and dark cycle. The animals were fed with standard pellet diet with water* ad libitum* in an animal house (Reg. number 1534/PO/a/11/CPCSEA) approved by the Committee for the Purpose of Control and Supervision on Experimental Animals (CPCSEA). The animals were acclimatized for ten days under laboratory conditions before carrying out the experiments.

### 2.4. Acute Toxicity and Selection of Test Doses

Earlier investigations indicate that* Channa striata* (Bloch.) aqueous extract is safe and nontoxic even at the dose of 8 g/kg or 100% w/v concentration [7, 8]. Doses of 30%, 40%, and 50% w/v were selected for the screening of antiulcer activity based on the report of Saleem et al. [10].

### 2.5. Evaluation of Antiulcer Activity

#### 2.5.1. Aspirin Induced Ulcerogenesis in Pylorus Ligated Rats

Aspirin induced ulcerogenesis in pylorus ligated rats model was used for the evaluation of antiulcer activity with slight modification. The animals were divided into five groups (*n* = 6). Group I served as negative control and received only vehicle. Groups II, III, and IV received aqueous extract of* Channa striata* (AECS) at 30%, 40%, and 50% w/v concentrations, respectively, orally at the volume of 10 mL/kg. Group V served as standard and was treated with standard drug (Ranitidine 50 mg/kg, p.o) [20]. Aspirin suspended in 1% CMC in water was administered orally at a dose of 500 mg/kg in 12-hour fasted rats [21]. The test extract and standard drug treatment were done 30 min prior to the administration of aspirin. After 30 min, the pyloric ligation surgery was performed. Four hours later, the animals were sacrificed by euthanasia.

*Collection and Measurement of Gastric Juice.* The stomachs were excised carefully keeping the esophagus closed. The stomachs were opened along the greater curvature, removing the luminal contents. The gastric contents were collected and centrifuged at 1000 rpm for 10 minutes. After centrifugation samples were decanted and the volume of gastric juice was noted and is expressed as mL/100 g body weight. The contents were subjected to analysis for free and total acidities.

*Determination of Gastric Juice pH.* One mL of supernatant liquid was diluted to 10 mL with distilled water. The pH of the solution was recorded with the help of digital pH meter.

*Estimation of Total and Free Acidities.* The above solution was titrated against 0.01 N NaOH using Topfer's reagent as indicator. The endpoint of the titration was when the solution turns orange in colour. The volume of NaOH was noted which corresponds to the free acidity. Further the titration was continued till the solution regained pink colour. The total volume of NaOH was noted, which corresponds to total acidity.

*Determination of Ulcer Index.* Mean ulcer score for each animal is expressed as ulcer index. The stomachs were washed with running water to see the ulcers in the glandular portion of the stomach. The number of ulcers per stomach was noted and the severity of the ulcers was scored microscopically with the help of hand lens (10x) and scoring was done as per Asru [22]:

 0 = Normal stomach
 0.5 = Red coloration
 1 = Spot ulcers
 1.5 = Haemorrhagic streaks
 2 = Ulcers >3 mm but <5 mm
 3 = Ulcers >5 mm.

The percentage of protection was calculated by the formula

(1) $$\begin{array}{c} \text{Percentage}\;\text{protection}\;=\;100\;-\;\left(\frac{\text{Ut}}{\text{Uc}}\times \;100\right), \end{array}$$

where Ut = ulcer index of the treated group and Uc = ulcer index of control group.

*Assessment of Oxidative Damage in Gastric Tissue.* After measuring the ulcer index, the stomachs were washed with 0.9% (w/v) NaCl, cut into small pieces, and homogenized with a glass homogenizer in ice-cold 0.15 M KCl solution to produce a 20% (w/v) homogenate. The homogenate was used for the determination of various biochemical parameters.

*(1) Estimation of Catalase.* Catalase containing sample is allowed to split H_2_O_2_ followed by adding dichromate/acetic acid mixture to stop the reaction. Dichromate in acetic acid is reduced first to unstable blue colored perchromic acid and finally to stable green colored chromic acetate in the presence of H_2_O_2_, which is measured colorimetrically at 570 nm. Thus chromic acetate measured gives the amount of freely available H_2_O_2_. To assay mixture containing 0.4 mL of 0.2 M H_2_O_2_ and 0.5 mL of 0.01 M phosphate buffer (pH 7), 0.1 mL of homogenate was added and mixed well. Into this, 2 mL of dichromate acetic acid solution was blown exactly after 1 min and kept in boiling water bath for 10 min. The absorbance of green colored chromic acetate formed was measured at 570 nm against reagent blank containing 0.4 mL of 0.2 M H_2_O_2_ and 0.5 mL of 0.01 M phosphate buffer (pH 7). The enzyme level is expressed as units/mg of tissue [23].

*(2) Estimation of Superoxide Dismutase.* The reaction involves generation of superoxide by pyrogallol auto-oxidation and the inhibition of superoxide dependent reduction of the tetrazolium dye MTT [3-(4-5 dimethyl thiazol 2-yl) 2,5-diphenyl tetrazolium bromide] to its formazan. The reaction is terminated by the addition of dimethyl sulfoxide (DMSO), which helps to solubilize the formazan formed. The color evolved is stable for many hours and is expressed as SOD units (one unit of SOD is the amount in mg of protein required to inhibit the MTT reduction by 50%). The reagents were added in the sample, control, and the blank as shown in Table 1.

The absorbance was read at 570 nm against distilled water (blank). Superoxide dismutase was expressed as SOD units/mg of tissue [24].

*(3) Estimation of Malondialdehyde.* Malondialdehyde, formed from the breakdown of polyunsaturated fatty acids, serves as a convenient index for determining the extent of peroxidation reaction. Malondialdehyde has been identified as the product of lipid peroxidation that reacts with thiobarbituric acid to give a red colour absorbing light maximally at 535 nm. One g of tissue sample with 10 mL of 0.2 M Tris HCl buffer (pH 7.2) was taken in a tissue homogenizer to get a 10% homogenate. 500 *μ*L of supernatant from the homogenate, 1 mL of 10% trichloroacetic acid, and 1 mL of 0.67% thiobarbituric acid were taken in a tightly stoppered tube. The tube was heated to boiling temperature for 45 min. After cooling the tube, the contents were centrifuged. The supernatant was read at 532 nm against blank. The concentration of test samples was obtained using molar extinction coefficient of MDA. The amount of MDA is expressed as number of moles of MDA/mg of tissue [25].

### 2.6. Statistical Analysis

Data obtained was analyzed by one-way ANOVA followed by Dunnett's multiple comparisons post hoc test using Graphpad Prism version 4.0, 32 bits for windows, Graphpad software, San Diego, California, USA (http://www.graphpad.com/). The values are expressed as mean ± standard error of mean (SEM). *P* < 0.05 was considered statistically significant.

## 3. Results 

### 3.1. Results of Aspirin Induced Ulcerogenesis in Pylorus Ligated Rats (Figure 2 and Table 2)

#### 3.1.1. Volume of Gastric Juice

AECS significantly (*P* < 0.05) decreased the volume of gastric juice at the dose of 40% w/v concentration. Further decrease in the output of gastric juice was observed with the treatment of higher concentration, that is, 50% w/v (*P* < 0.01). However, the least test dose (30% w/v) used in the study did not show any significant antisecretory effect (as shown in Figure 2 and Table 2).

#### 3.1.2. pH of Gastric Juice

Treatment with the standard drug (Ranitidine 50 mg/kg) significantly (*P* < 0.01) raised the pH from 3.83 ± 0.10 (negative control) to 4.95 ± 0.16, while all the concentrations of AECS failed to elevate pH of the gastric juice (as shown in Figure 2 and Table 2).

#### 3.1.3. Free and Total Acidities

Likewise 30% and 40% w/v solutions of AECS did not alter free and total acidities. A slight (*P* < 0.05) decrease in free and total acidities was observed with the treatment of 50% w/v AECS solution against the significant decrease in free and total acidities by the standard drug with *P* < 0.01 and *P* < 0.001, respectively (as shown in Figure 2 and Table 2).

#### 3.1.4. Ulcer Index (UI)

All the test doses of AECS solutions (30%, 40%, and 50% w/v) significantly decreased the ulcer index (*P* value ranging from 0.01 to 0.001). The decrease in ulcer index in the AECS (50% w/v) treated group is comparable with that of the standard drug. A dose related increase in percentage protection was observed. The percentage of inhibition for 40% and 50% AECS was found to be 36.54% and 47.88%, respectively, while the standard showed 53.46% (as shown in Figure 2 and Table 2).

#### 3.1.5. Catalase (CAT)

A dose dependent increase was noticed with the treatment of AECS with *P* value ranging from 0.01 to 0.001, except the lowest test dose used in the experiment. The results of standard and 40% w/v AECS were found to be similar, while 50% AECS exhibited maximum increase in the level of CAT with *P* < 0.001 (as shown in Figure 2 and Table 2).

#### 3.1.6. Superoxide Dismutase (SOD)

AECS at 30% and 40% w/v concentrations did not cause significant increase in the level of superoxide dismutase whilst 50% w/v AECS raised the level of SOD with *P* < 0.05. The treatment with the standard displayed the most significant rise with *P* < 0.01 (as shown in Figure 2 and Table 2).

#### 3.1.7. Malondialdehyde (MDA)

The standard drug and all the test doses of AECS significantly decreased the formation of malondialdehyde, an end product of lipid peroxidation. A potent as well as consistent decrease in the level of MDA (*P* < 0.001) was observed. The decrease in MDA was proportionate to the increasing test doses; further the effect of all the concentrations of AECS was more profound than the standard. This highlights the gastroprotective potential of AECS against lipid peroxidation of gastric mucosa. The results are displayed in Figure 2 and Table 2.

#### 3.1.8. Macroscopy of Stomachs

**Figure 1:**
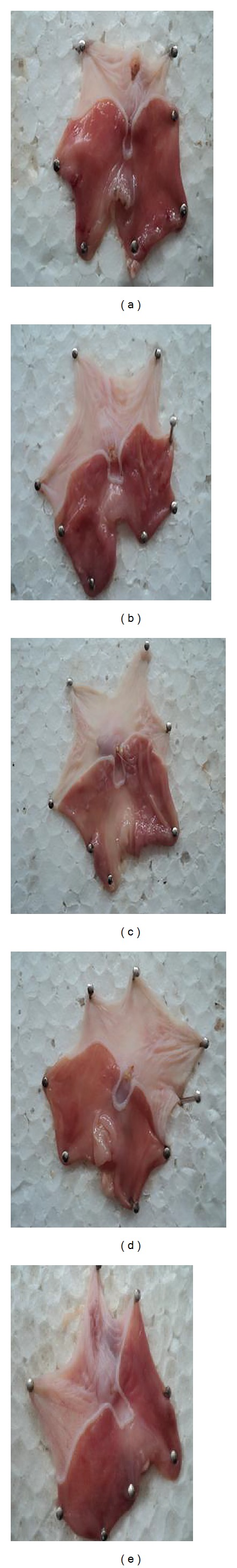
Photos of rat's stomachs subjected to aspirin induced ulcerogenesis in pyloric ligation model. Note: (a) negative control; (b) Test-I (AECS 30% w/v); (c) Test-II (AECS 40% w/v); (d) Test-III (AECS 50% w/v); and (e) standard (Ranitidine 50 mg/kg).

**Figure 2:**
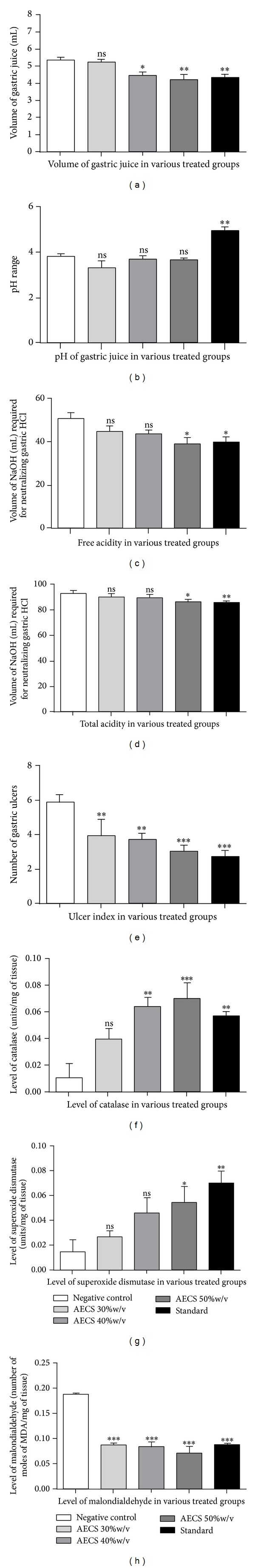
Graphs showing effect of AECS and standard drug on various gastric parameters.

- *Negative Control. *The stomach shows red coloration, haemorrhage, hyperaemia, one spot ulcer, two ulcers with diameter in range of 3–5 mm, and two ulcers greater than 5 mm.
- *AECS (30% w/v).* Red coloration and four spot ulcers are seen in the stomach.
- *AECS (40% w/v).* The stomach coloration is normal but shows two spot ulcers.
- *AECS (50% w/v).* Erythema, an inflammatory sign, can be observed in the stomach with complete absence of ulcers.
- *Standard. *The stomach reveals partial red coloration with no ulcers. The images are shown in Figure 1.

## 4. Discussion

*Channa striata *contains unsaturated fatty acids (UFA) and essential amino acids (AA) that stimulate and promote healing of wounds [3, 18, 19, 26].* C. striata *contains alanine, arginine, aspartic acid, glutamic acid, glycine, leucine, and proline [27]. An earlier report of Gam et al. reported lysine, threonine, and valine as the other most abundant amino acids in* C. striata *[28]. Some of these amino acids are the integral components of gastric mucous [29]. Furthermore some of these amino acids are known to have significant antioxidant properties particularly with linoleic acid [30].

*C. striata* fresh fillet also contains high amount of glycine which is one of the important components responsible for the formation of collagen in the various tissues of human body [3, 27, 31].* C. striata *treatment promotes remodeling of collagen through the synthesis of inter- and intramolecular protein cross-linking. This action strengthens the body tissues and prevents further degradation [1, 15]. Glycine is the amino acid present in* C. striata *in the highest concentration. Glycine is important in healing process as it is one of the major components of human tissue collagen. It promotes tissue repair synergistically by forming a polypeptide with other essential amino acids like alanine, arginine, isoleucine, phenylalanine, proline, and serine [32].

Arginine plays critical and multiple roles in wound healing process. It stimulates the release of hormones like growth hormone from pituitary and insulin from pancreas [33]. In postinjury catabolic state, it decreases the urinary nitrogen loss to regulate nitrogen balance [34]. This amino acid is also a substrate for two integral enzymes—nitric oxide synthetase (NOS) and arginase. Arginine is metabolized in wounds by the action of enzyme arginase abundantly present in wound fluid [35]. Arginine produces hydroxyproline, an important component (9.1% of the total amino acid residue) of collagen [36]. Aspartic acid an excitatory amino acid that is involved in antioxidant mechanism is also found in high amount in* C. striata *[37].

The dominant fatty acids in* C. striata *are palmitic acid (C16: 0), stearic acid (C18: 0), oleic acid (C18: 1n-9), and linoleic acid (C18: 2n-6) [27]. Polyunsaturated fatty acids (PUFA) components (arachidonic and linoleic acids) of* C. striata *decrease serum cholesterol and triglycerides levels and inhibit clotting of blood [38]. This action can facilitate the supply of blood to the gastric tissue countering the obstruction, one of the etiological factors in the pathogenesis of peptic ulcers. Further, the report of Mat Jais et al. suggests the high compositions of fatty acids and certain amino acids as responsible agents for the healing effects of* C. striata* [3]. The oleic and stearic acids are reported to attenuate polymorphonuclear leukocytes activity and influence membrane fluidity, consequently suppressing inflammatory processes [27]. Arachidonic acid which is a precursor of prostaglandin is found in* C. striata* in considerable amounts. The prostaglandins play a major role in growth of tissue and wound healing [39–41].

Furthermore, Huang et al. have reported that arachidonylglycine (a lipoamino acid formed by the conjugation of arachidonic acid and glycine) suppresses edema and pain [42]. This can relieve or minimize the gastric distress that accompanies peptic ulceration. Vitamin-A (Retinol) an essential factor for wound healing is also present in high concentration in* C. striata* [3, 43].

*C. striata* extract has also demonstrated inhibitory effects on* H. pylori *[44]. Besides promising results as an antibacterial and antifungal agent against certain strains,* C. striata *extracts exhibited antibacterial activity in various studies against wide range of bacteria like* Staphylococcus aureus* [13],* Aeromonas hydrophila* and* Pseudomonas aeruginosa* [14],* Bacillus subtilis*,* Klebsiella pneumoniae, *and* Pseudomonas aeruginosa* [45].* C. striata* extracts also demonstrated antifungal activities against* Aleurisma keratinophilum*,* Botrytis pyramidalis*,* Cordyceps militaris*,* Neurospora crassa, *and* Paecilomyces fumosoroseus* [13].

The beneficial effects of aqueous extract of* Channa striata* (AECS) were observed at the higher test doses. The ineffectiveness of low dose of AECS (30% w/v) could be due to the presence of bioactive compounds in quantities lesser than the minimum effective dose. Therefore, there is a need for a similar study at higher concentrations to evaluate gastroprotective potential of AECS appropriately and justify the observations of the present investigation properly.* C. striata *is a nutraceutical agent and well tolerable even at the higher concentrations; the prospect of increasing the test doses in the future studies does exist. The test doses can be increased up to 100% w/v concentration [8] or 8 g/kg [7] as used by them in their studies. Additionally, in view of report by Dahlan-Daud et al., [27] on the chemical composition of various fractions of* C. striata*, it is expected that a similar study using the other bioactive fractions like Haruan Commercial Essence and Lower Phase of Haruan Traditional Extract would give more meaningful results.

## 5. Conclusion

The present study reveals that AECS is devoid of gastric acid neutralizing effect but possesses potent antisecretory and antiulcer properties. The observed pharmacological action can be attributed to the essential amino acids and unsaturated fatty acids present in the extract. Further, this is the first study that highlights antioxidant potential of AECS in an* in vivo* experiment and indicates antioxidant activity as one of the probable mechanisms for the gastroprotective effect of AECS. It can be concluded that AECS possesses antisecretory and antiulcer properties, particularly at 40% and 50% w/v concentrations.

## Figures and Tables

**Table 1:** Details of various reagents and their quantities added to sample, control, and blank during the estimation of superoxide dismutase.

Reagents	Sample	Control	Blank (duplicate)
PBS	0.65 mL	0.65 mL	0.65 mL
MTT	30 *μ*L	30 *μ*L	30 *μ*L
Homogenate	10 *μ*L	—	—
Pyrogallol	75 *μ*L	75 *μ*L	75 *μ*L

The sample, control, and blank were incubated for 5 min at room temperature			
DMSO	0.75	0.75	0.75

**Table 2:** Results of aspirin induced ulcerogenesis in pyloric ligation model.

Parameters	Various groups				
	Negative control	Ranitidine	Aqueous extract of *Channa striata *		
		50 mg/kg	30% w/v	40% w/v	50% w/v
Volume of gastric juice (mL)	5.43 ± 0.22	4.36 ± 0.17**	5.26 ± 0.16^ns^	4.53 ± 0.16*	4.23 ± 0.32**
pH	3.83 ± 0.108	4.95 ± 0.16**	3.33 ± 0.33^ns^	3.68 ± 0.18^ns^	3.65 ± 0.084^ns^
Free acidity	51.00 ± 2.85	40.00 ± 2.47*	45.00 ± 2.72^ns^	44.00 ± 1.59^ns^	39.33 ± 2.97*
Total acidity	93.17 ± 1.86	85.50 ± 1.40**	90.50 ± 1.72^ns^	90.17 ± 1.74^ns^	86.67 ± 1.20*
Ulcer index	5.91 ± 0.45	2.75 ± 0.38***	4.00 ± 0.36**	3.75 ± 0.38**	3.08 ± 0.35***
Percentage of inhibition	—	53.46%	32.31%	36.54%	47.88%
Catalase (units/mg of tissue)	0.011 ± 0.011	0.059 ± 0.0032**	0.041 ± 0.0079^ns^	0.066 ± 0.0064**	0.072 ± 0.012***
Superoxide dismutase (units/mg of tissue)	0.015 ± 0.010	0.071 ± 0.0094**	0.027 ± 0.0050^ns^	0.047 ± 0.012^ns^	0.055 ± 0.013*
Malondialdehyde (moles/mg of tissue)	0.190 ± 0.0012	0.089 ± 0.0013^∗∗∗^	0.088 ± 0.0034^∗∗∗^	0.085 ± 0.0078^∗∗∗^	0.072 ± 0.013***

Note: sample size (*n*) = 6 rats per group. Data is expressed as mean ± standard error of mean and standard deviation in parenthesis. *P < 0.05, **P < 0.01, ***P < 0.001 and ns nonsignificant versus negative control (on statistical analysis with ANOVA, followed by Dunnett's multiple comparison post hoc test).
